# A Method for the Precise Determination of the Intramolecular Potential Energy Surface on the Basis of Microwave and Submillimeter-Wave Spectra: Diatomic Molecules as a Relevant Illustration

**DOI:** 10.3390/ijms26020658

**Published:** 2025-01-14

**Authors:** Oleg Ulenikov, Elena Bekhtereva, Olga Gromova, Sergei Sidko, Sigurd Bauerecker

**Affiliations:** 1 Research School of High-Energy Physics, National Research Tomsk Polytechnic University, Tomsk 634050, Russia; lane_bes@mail.ru (E.B.);; 2 Institut für Physikalische und Theoretische Chemie, Technische Universität Braunschweig, D-38106 Braunschweig, Germany; s.bauerecker@tu-bs.de

**Keywords:** intramolecular potential energy surface, diatomic molecule, basic parameters of HCl molecule

## Abstract

A new method for the precise semiempirical determination of the basic parameters (structural parameters and parameters of the intramolecular potential energy surface, PES) of a molecule on the basis of highly accurate experimental data from the microwave and submillimeter-wave regions is suggested. The options and advantages of this method in comparison with the other methods of molecular PES determination are discussed using a diatomic molecule as an appropriate illustration. The HCl molecule is exploited as a suitable example. It is shown with this example that the use of a very limited number (ten for H^35^Cl and five for D^35^Cl) of submillimeter-wave line positions allows one to determine the values of the equilibrium rotational parameter, harmonic frequency, and anharmonic coefficients of the third, fourth, and fifth order with accuracy of 0.01%, 0.01%, 0.01%, 2.1%, and 10.1%, respectively, in comparison with the analogous results obtained from extensive infrared studies.

## 1. Introduction and General Description of the Problem

For a long time, precise knowledge of the intramolecular potential surface (PES) of a molecule was (and continues to be) one of the most important problems in physical chemistry. In modern physical chemistry, two main methods are usually used for the determination of the parameters of the intramolecular potential functions of several molecules. One of them is the method of direct quantum mechanical PES calculation (exploiting different modifications of the ab initio method). Here, it is not possible to cover the huge quantity of books and original papers that have been prepared in this direction, and we mention only a few of them, i.e., Refs. [1,2,3,4,5,6,7,8,9,10,11]. An alternative way to derive a molecular PES is the so-called semiempirical method, which is based on accurate knowledge of the line positions of a studied molecule in sufficiently broad infrared (IR) spectral ranges and the possibility to produce a set of relations in analytical form that is able to connect the required PES parameters with spectroscopic parameters obtained from experimental data (such as harmonic frequencies $\omega_{\lambda }$, anharmonic coefficients $x_{\lambda \mu }$, and resonance interaction parameters); see, e.g., [12,13,14]. The main advantage of the first (ab initio) method is that it allows for the more (for small molecules) or less (for large molecules) correct qualitative description of a molecular PES across a wide range of changes in the bond length and interbond angle. At the same time, to date, even with the use of modern supercomputers and even for the smallest molecules (we do not discuss molecules with more than four nuclei), the accuracy of the prediction of both the line positions and the numerical values of the PES parameters is considerably worse in comparison with the accuracy of modern experiments in the infrared (IR) region or in the microwave (MW) and submillimeter-wave (SMW) regions. At the same time, from a quantitative point of view, the semiempirical method gives considerably better results in the vicinity of the equilibrium configuration of a molecule in comparison with the ab initio methods. The best results, of course, can be obtained with the use of the comprehensive approach, which consists of ab initio calculations with the further calibration of the obtained results using data derived from semiempirical analysis. In any case, the precise determination of the semiempirical intramolecular PES for a concrete molecule is a very important and relevant problem.

Regarding the latter problem (semiempirical PES determination on the basis of highly accurate experimental infrared data), it is complicated by at least two factors. The first one is the following: for the realization of the semiempirical procedure, even for the simplest polyatomic (tri-/tetra-atomic) molecule (for example, for the determination of even the fourth-order anharmonic parameters of a molecular PES), it is necessary to have highly accurate experimental information about a large number of not only the fundamental and first overtone ro-vibrational bands but for a number of high-order overtone and combinational bands as well. However, especially if one takes into account the presence of numerous resonance interactions and so-called “forbidden” bands for essentially any polyatomic molecule, this has never happened in reality. Another (perhaps even more serious) problem is the following: to realize the highly accurate semiempirical determination of a molecular PES, it is necessary to have (or determine) corresponding analytical relations/formulas that connect the spectroscopic parameters derived from the analysis of the experimental data with the desired intramolecular PES parameters. The derivation of such formulas necessitates the use of high-order corrections from operator perturbation theory (see, e.g., [12,15,16,17]), which is an extremely complicated task if we consider high-order vibrational parameters and the need for the consideration of different types of resonance interaction parameters.

In this paper, we present some considerations that will allow us to solve the problem of the semiempirical determination of molecular PES parameters (up to fifth- or sixth-order force parameters for simple molecules, or up to fourth- or fifth-order force parameters for molecules with five or more nuclei) on the basis of highly accurate experimental data from the microwave and submillimeter-wave regions. Such a possibility is based on three main points (two of which have obvious advantages in comparison with the analogous method based on the use of IR data). The first is the very high accuracy of microwave and submillimeter-wave data, which gives the possibility to determine the rotational parameters of a molecule with accuracy of up to eight or nine significant digits; the Δ– and H–centrifugal parameters can be determined from experimental data with accuracy of up to six to seven or four to five significant digits, respectively. The second advantage is in the considerably simpler algorithm used to derive formulas/relations that connect the necessary “experimental” spectroscopic parameters with even the high-order force parameters of a molecular PES. It is also important that, in this case, the problems of both resonance interactions and the need to consider highly excited vibrational states are absent. Knowledge of the results and statements of isotopic substitution theory (see, e.g., [18,19] and references therein) is the third helpful point, because only the joint consideration of many different isotopologues of a molecule can offer the possibility to solve this problem. Moreover, in this respect, it should be noted that, contrary to the use of infrared experimental data, (1) even the experimental recording of a small number of lines of a few isotopologues gives the possibility to obtain the correct values of its spectroscopic parameters, which can be used in further analysis, and (2) the number of such different isotopologues can be large enough (as an illustration, see Ref. [20], where the microwave spectra of twelve different species of ethyl chloride and ten different species of vinyl chloride were recorded, with the goal of the determination of the bond lengths and interbond angles of these molecules).

As an “instrument” to illustrate the possibilities and peculiarities of the suggested procedure, in this paper, we consider the simplest molecule, namely a diatomic molecule. As the next step in this project’s realization, some asymmetric top molecules (several triatomic XY_2_ (C_2*v*_ symmetry) and the CH_2_Cl_2_ one) will be considered in the near future, and their force PES parameters (up to at least the fifth order) will be determined.

## 2. The Hamiltonian of a Diatomic Molecule and the Spectroscopic Parameters

Taking into account that we plan to use the discussed method/procedure to solve the problem of PES determination for polyatomic molecules, we present the rotation–vibration Hamiltonian of a diatomic molecule in a form that, on the one hand, is as compatible as possible with the Hamiltonian of any polyatomic molecule (see [14]), and, on the other hand, is close to the classical Hamiltonian of a diatomic molecule from [21]:

(1) $$\begin{array}{c} H=H_{\mathrm{harm}}+H_{\mathrm{anh}}+H_{v\text{-}r}, \end{array}$$

where

(2) $$\begin{array}{c} H_{\mathrm{harm}}=\frac{\omega }{2}\left(p^{2}+q^{2}\right) \end{array}$$

is the Hamiltonian of the harmonic oscillator, and the anharmonic part of an intramolecular PES is taken as

(3) $$\begin{array}{c} H_{\mathrm{anh}}=\sum_{n=3}^{\infty }k_{n}q^{n}. \end{array}$$

(4) $$\begin{array}{c} H_{v\text{-}r}=\left(B_{e}+\sum_{m=1}b_{m}q^{m}\right)\sum_{\alpha }J_{\alpha }^{2}\;\;\left(\alpha =x,y,z\right) \end{array}$$

is the operator that describes the interaction between vibration and rotation in a molecule. In Equations (2)–(4), *p* and *q* are operators of dimensionless vibrational coordinates and impulse $p=-i\frac{\partial }{\partial q}$; see Ref. [12]. $b_{0}=\omega^{2}/4B_{e}$; ω is the harmonic frequency (in cm^−1^); $B_{e}=\frac{\hbar }{4\pi cr_{e}^{2}\mu }$$\left(\mu =\frac{m_{1}m_{2}}{m_{1}+m_{2}}\right)$ is the equilibrium rotational parameter (also in cm^−1^); $k_{n}$ (i=3,4,5,…) are the molecular PES parameters that one would like to obtain; $J_{\alpha }$ are components of the molecular angular momentum operator; and

(5) $$\begin{array}{c} b_{m}=\left(m+1\right)B_{e}{\left(\frac{2B_{e}}{\omega }\right)}^{m/2} \end{array}$$

are parameters that depend only on the masses of the nuclei $m_{1}$ and $m_{2}$ and the equilibrium bond length $r_{e}$.

In accordance with the classical paper by Dunham [21], the eigenvalues $E_{vJ}$ of the Hamiltonian, as in Equation (1), can be expressed in a very simple form as

(6) $$\begin{array}{c} E_{vJ}=\sum_{ij}Y_{ij}{\left(v+\frac{1}{2}\right)}^{i}{\left\{J(J+1)\right\}}^{j}, \end{array}$$

where v=0,1,2,… and J=0,1,2,… are vibrational and rotational quantum numbers, respectively. The values $Y_{ij}$ are the spectroscopic parameters of a diatomic molecule, which are analogous to the spectroscopic parameters of a polyatomic molecule as discussed in Section 1. They can be determined from the analysis of highly accurate MW/SMW experimental data and can also be obtained as analytical relations that connect them with the structural (in the case of a diatomic molecule, it is the only parameter, namely the equilibrium bond length $r_{e}$) and force PES parameters (in the present case, they are the ω, $k_{3}$, $k_{4}$, $k_{5}$, etc., parameters). For a diatomic molecule, the determination of such types of relations, even with the use of high orders in perturbation theory, is a rather simple procedure because only one vibrational coordinate is present in a molecule. As a consequence, we do not discuss here the problem of the determination of the corresponding necessary relations and note only that such relations that are important for the purposes of the present analysis of the MW and SMW experimental data are reproduced from [21] and shown in Appendix A.

## 3. Determination of the Structural $r_{e}$ and Force ω and $a_{n}$ Parameters from the MW and SMW Experimental Data

Let us consider, step by step, the above-discussed procedure for the determination of the structural and force PES parameters with the diatomic molecule as an illustration. We believe that one of the three main points mentioned in Section 1 (namely the presence of relations that connect the values of the experimentally obtained spectroscopic parameters with the structural and force PES parameters; see Appendix A) is realized. Regarding the second point (the presence of highly accurate experimental data on the MW and/or SMW regions), it is necessary that the molecular MW and/or SMW spectra are determined by transitions within the same vibrational state. In this case, it is not difficult to show that the rotational–vibrational energies $E_{vJ}$ of the vibrational state (v) can be expressed in the following form:

(7) $$\begin{array}{c} E_{vJ}=B^{\left(v\right)}J\left(J+1\right)+D^{\left(v\right)}{\left\{J(J+1)\right\}}^{2}+H^{\left(v\right)}{\left\{J(J+1)\right\}}^{3}+\ldots , \end{array}$$

where the rotational parameters $B^{\left(v\right)}$ and centrifugal distortion parameters $D^{\left(v\right)}$, $H^{\left(v\right)}$, etc., are determined as

(8) $$\begin{array}{c} B^{\left(v\right)}=\sum_{i=0}^{\infty }Y_{i1}{\left(v+\frac{1}{2}\right)}^{i}, \end{array}$$

(9) $$\begin{array}{c} D^{\left(v\right)}=\sum_{i=0}^{\infty }Y_{i2}{\left(v+\frac{1}{2}\right)}^{i}, \end{array}$$

(10) $$\begin{array}{c} H^{\left(v\right)}=\sum_{i=0}^{\infty }Y_{i3}{\left(v+\frac{1}{2}\right)}^{i}, \end{array}$$

etc. As a consequence, from the MW and/or SMW experimental spectra, one can obtain three (or even four or five, in some cases) highly accurate combinations of spectroscopic parameters, as in Equations (8)–(10), for the separate vibrational states of a diatomic molecule. Concerning a polyatomic molecule, evidently, one can expect that analogous considerations will lead to the three $A^{\left(v\right)}$, $B^{\left(v\right)}$, and $C^{\left(v\right)}$ rotational parameters (or combinations of corresponding $Y_{ij}$ parameters) for an asymmetric top molecule or two rotational parameters for a symmetric top molecule. In turn, one could expect that five and seven of the quartic and sextic centrifugal distortion parameters of the asymmetric top molecule (five and seven combinations analogous to the $Y_{ij}$ parameters), or three and four quartic and sextic centrifugal distortion parameters of the symmetric top molecule, could be determined with high accuracy from the MW and SMW spectra of a concrete vibrational state of a molecule. In the following, we discuss how such information can be used for the precise determination of the force PES parameters, with the HCl molecule as an illustration.

Returning to the Hamiltonian, i.e., Equations (1)–(4), one can see that the unknown parameters in the Hamiltonian are the equilibrium length $r_{e}$ (or equilibrium rotational parameter $B_{e}$), the harmonic frequency ω, and the anharmonic coefficients $k_{3},k_{4},k_{5},\ldots$. If we look now at the spectroscopic parameters $Y_{ij}$ in Appendix A, then we can conclude that the parameter $Y_{01}$ allows us to determine the $r_{e}$ (or $B_{e}$) value, while the parameter $Y_{02}$ gives the possibility to determine the value of the harmonic frequency ω (another point for discussion is the analysis of the accuracy of the determination of the ω value from the centrifugal $Y_{02}$ parameter). In turn, the $Y_{11}$ and $Y_{03}$ parameters allow us to determine the value of the $k_{3}$ force parameter; meanwhile, the values of the $Y_{12}$, $Y_{21}$, and $Y_{13}$ parameters can be used for the determination of the $k_{4}$, $k_{5}$, etc., parameters. As will be shown, the accuracy of the MW and SMW data is high enough for the determination of the required parameters. Only one problem remains: the number of such required parameters is larger than the number of the three parameters $B^{\left(v\right)}$, $D^{\left(v\right)}$, and $H^{\left(v\right)}$ obtained from the experimental data.

To solve the latter problem, for a polyatomic molecule, one can use the results and conclusions of isotopic substitution theory (see, e.g., [18,19]). In the case of a diatomic molecule, all possible isotopic relations can be derived from the following simple analysis. Following the general consideration of an arbitrary linear molecule, we use the vibration–rotation Hamiltonian (the diatomic molecule can be considered as the simplest example of such molecules) [22]. We also take into account that (in the Born–Oppenheimer approximation [23]), the vibrational part $H_{vibr}=H_{\mathrm{harm}}+H_{\mathrm{anh}}$ of the Hamiltonian (see Equation (1)), written in the $\Delta r=(r-r_{e})$ coordinates (analogously to [13]),

(11) $$\begin{array}{c} H_{vibr}=\frac{1}{2}f_{2}\Delta r^{2}+\sum_{n=3}\frac{f_{n}}{r_{e}^{\left(n-2\right)}}\Delta r^{n}, \end{array}$$

is the same for any isotopologue of a molecule (note that all $f_{n}$ parameters in Equation (11) have a dimension of md/Å). In this case, it is not difficult to show that the force parameters $k_{n}$ from Equation (4) (which, like the dimensionless vibrational *q* coordinates, are different for different isotopologues) are connected with the “universal” force $f_{n}$ parameters with the help of the following relations:

(12) $$\begin{array}{c} \omega =\frac{1}{2\pi c}{\left(\frac{f_{2}}{\mu }\right)}^{1/2}, \end{array}$$

(13) $$\begin{array}{c} k_{3}=\frac{\sqrt{2}}{3}{\left(\frac{B_{e}}{\omega }\right)}^{3/2}\left(\frac{f_{3}r_{e}^{2}}{2\pi c\hbar }\right), \end{array}$$

(14) $$\begin{array}{c} k_{4}=4{\left(\frac{B_{e}}{\omega }\right)}^{2}\left(\frac{f_{4}r_{e}^{2}}{2\pi c\hbar }\right), \end{array}$$

(15) $$\begin{array}{c} k_{5}=4\sqrt{2}{\left(\frac{B_{e}}{\omega }\right)}^{5/2}\left(\frac{f_{5}r_{e}^{2}}{2\pi c\hbar }\right), \end{array}$$

(16) $$\begin{array}{c} k_{6}=8{\left(\frac{B_{e}}{\omega }\right)}^{3}\left(\frac{f_{6}r_{e}^{2}}{2\pi c\hbar }\right), \end{array}$$

etc., or, in a general case,

(17) $$\begin{array}{c} k_{n}=2^{n/2}{\left(\frac{B_{e}}{\omega }\right)}^{n/2}\left(\frac{f_{n}r_{e}^{2}}{2\pi c\hbar }\right). \end{array}$$

As one can see, the force $k_{n}$ parameters of different isotopologues differ from each other only by the part ${\left(\frac{B_{e}}{\omega }\right)}^{n/2}$. As a consequence, if one takes the relations from Appendix A into account, it is possible to obtain very simple relations between the spectroscopic parameters $Y_{ij}$ and ${\tilde{Y}}_{ij}$ of any two isotopologues of a molecule in the following form:

(18) $$\begin{array}{c} {\tilde{Y}}_{ij}={\left(\frac{\mu }{\tilde{\mu }}\right)}^{\left(i/2+j\right)}Y_{ij}. \end{array}$$

The presence of very simple relations between $Y_{ij}$ and the spectroscopic parameters for diatomic molecules has dual significance in the discussion of the problem for diatomic molecules. On the one hand, the presence of such simple relations is very positive if one wishes to predict the values of the spectroscopic parameters of one isotopologue on the basis of information about the known values of the corresponding spectroscopic parameters of another isotopologue. On the other hand, to address the problem discussed in the present study, the presence of simple relations (Equation (17)) has both positive and negative consequences. It is beneficial that (by deriving the values of the three $B^{\left(v\right)}$, $D^{\left(v\right)}$, and $H^{\left(v\right)}$ parameters, using Equations (8)–(10), from the highly accurate MW/SMW experimental data for some different isotopologues and using the relations in Equation (18)) one can construct additional equations for the separate determination of the spectroscopic parameters on the right-hand sides of Equations (8)–(10) and, as a consequence, to determine both $r_{e}$, ω, and higher-order force PES parameters. On the other hand, the presence of simple relations (Equation (18)) leads to a situation in which, as the analysis shows, the corresponding right-hand sides of Equations (8)–(10) for isotopologues with close values of the reduced masses μ and $\tilde{\mu }$ become almost dependent. The latter can lead to close-to-zero values of the determinants of the so-called “dispersion matrix **V**” [24] and to the impossibility of the separate determination of the parameters on the right-hand sides of Equations (8)–(10). For polyatomic molecules, simple relations, analogous to Equation (18), are absent as a rule. For this reason, one can expect that, for polyatomic molecules, it will be possible to construct a suitable set of independent equations for the determination of the necessary parameters.

## 4. Determination of the Structural $r_{e}$ and Force ω and $k_{n}$ Parameters from the MW and SMW Experimental Data: The HCl Molecule

Let us apply the above-discussed procedure to the analysis of the real HCl molecule and compare the obtained results with the corresponding information obtained on the basis of the IR experimental data; see Ref. [25]. As the necessary initial information for the present analysis, MW/SMW experimental data for the ground state transitions of four different HCl isotopologues (H^35^Cl, H^37^Cl, D^35^Cl, and D^37^Cl) were used from Refs. [26,27] (for the convenience of the reader, the corresponding information from [26,27] is reproduced in Table 1). All data from Table 1 were used then in the weighted fit of the $B^{\left(v\right)}$, $D^{\left(v\right)}$, and $H^{\left(v\right)}$ ground state parameters of these four isotopologues. The obtained values are shown in Table 2, together with the values of their 1σ statistical confidence intervals obtained from the fit (the latter are shown in parentheses). In turn, the data from Table 2 and Equation (18) can be used for the determination of the separate $Y_{i1}$ (from Equation (8) for the four different isotopologues), $Y_{i2}$, or $Y_{i3}$ (again for the different Equations (9) or (10)) spectroscopic parameters. Unfortunately, as noted above, the four equations (8) practically coincide in pairs for the H^*M*^Cl and D^*M*^Cl (M=35,37) species (so, in reality, only two of the four relations of the Equation (8) type can be considered independent; the same can be said about Equations (9) and (10)). As a consequence, there are only two truly independent equations for the $B^{\left(v\right)}–$type parameters, as well as for the $D^{\left(v\right)}$ and $H^{\left(v\right)}$ parameters. Evidently, the third independent equation for any of the parameters (using Equations (8)–(10)) could be constructed if one had experimental information about the MW/SMW transitions—for example, for one of the T^*M*^Cl species (T here is the tritium isotope of the hydrogen nucleus). In any case, if one takes into account that $Y_{0j}\gg Y_{1j}\gg Y_{2j}\gg \ldots$ (j=1,2,3,…), it is clear that the values of the $Y_{01}$, $Y_{11}$, $Y_{02}$, $Y_{12}$, $Y_{03}$, and $Y_{13}$ parameters can be obtained from the experimental values of the parameters from Table 2 (they are presented in column 2 of Table 3). In turn, the latter allows us (if one takes into account the relations from Appendix A) to determine five basic parameters of the HCl molecule, such as $B_{e}$ (or $r_{e}$), ω, $a_{1}$, $a_{2}$, and $a_{3}$; see column 2 of Table 4. For comparison, column 4 of Table 4 presents the values of the corresponding parameters that were obtained on the basis of the IR experimental data [25]. One can see good correlations between both sets of parameters. Note that the addition of minimal MW/SMW experimental data would give the possibility to determine, with high accuracy, the corresponding $B^{\left(v\right)}$, $D^{\left(v\right)}$, and $H^{\left(v\right)}$ parameters of TCl and, as a consequence, to obtain the $Y_{21}$, $Y_{22}$, and $Y_{23}$ values. In turn, the latter would allow us to obtain the values of the $a_{4}$ and $a_{5}$ force parameters.

As discussed above, through the addition of the MW/SMW experimental data for the TCl molecule, it is possible to determine the values of even higher-order force parameters. In this case, evidently, the values of the $Y_{01}$, $Y_{11}$, $Y_{02}$, $Y_{12}$, $Y_{03}$, and $Y_{13}$ parameters (as a consequence of the required corresponding basic parameters) can be improved. To check and confirm this statement, we performed the following analysis. Because the value of the $a_{4}$ parameter was estimated from the analysis of IR data [25], one can estimate the values of the parameters $Y_{21}$ and $Y_{22}$ in Equations (8) and (9) for all four discussed isotopologues and to improve the values of the corresponding parameters on the left-hand sides of Equations (8) and (9). As a consequence, one can expect that the values of the $Y_{01}$, $Y_{11}$, $Y_{02}$, and $Y_{12}$ parameters (as well as of the corresponding basic parameters) could be improved. The results of these corrections are shown in column 3 of Table 4. One can see that the obtained results are closer to the corresponding values in column 4 in comparison with the results of the first analysis given in column 2.

It is interesting to consider the derived force constant in the form of the $f_{n}$ parameters from Equation (11), which is widely used in IR spectroscopy. The comparison of the HCl PES in the form of Equation (11) with the corresponding PES in the form of Equation (2) from Ref. [21] allows one to directly obtain the values of the $f_{n}$ parameters in Equation (11) as $f_{2}=5.1458$ md/Å, $f_{3}=-6.0878$ md/Å, $f_{4}=9.2504$ md/Å, and $f_{5}=-10.8379$ md/Å.

## 5. Discussion

From the above considerations, one can conclude that the suggested method for the determination of the basic molecular parameters on the basis of the experimental MW and SMW data is correct and considerably efficient. Furthermore, taking into account the statements above about polyatomic molecules, this method gives the possibility for the determination of considerably more extensive and correct quantitative information about the intramolecular force PES parameters in comparison with the current analogous semiempirical method, which is based on IR experimental data, or with the results obtained on the basis of “ab initio” calculations. As was mentioned above, we present (after developing the necessary analytical formulas that connect the rotational parameters and quartic and sextic centrifugal distortion parameters obtained from the MW and SMW spectra with the basic PES parameters) the above-discussed procedure for application to asymmetric top molecules of the XY_2_ ($C_{2v}$ symmetry) type and to the CH_2_Cl_2_ molecule. In this case, it would be suitable to discuss here the potential possibilities of the suggested method with, for example, the XY_2_ molecule as an illustration. First of all, it is interesting and important to estimate and compare the general number of required basic parameters of a molecule and the possible number of rotational and centrifugal distortion coefficients that can be determined from the highly accurate MW and SMW experimental data.

For the ground vibrational state of an asymmetric top molecule, at least three rotational parameters, five Δ–type, and seven H–type centrifugal distortion parameters can be determined with high accuracy (i.e., 15 parameters for the separate vibrational states of a molecule). In this case, the unknown required parameters, e.g., for the XY_2_, $C_{2v}$ symmetry molecule, are two equilibrium structural parameters, $r_{e}$ and $\alpha_{e}$; three harmonic frequencies $\omega_{\lambda }$ (λ=1,2,3); and one of two Coriolis coefficients $\zeta_{12}^{y}$ and $\zeta_{13}^{y}$; see, e.g., [28]. We also require six cubic $k_{\lambda \mu \nu }$, nine quartic $k_{\lambda \mu \nu \xi }$, twelve pentic $k_{\lambda \mu \nu \xi \varsigma }$, and 19 sextic $k_{\lambda \mu \nu \xi \varsigma \chi }$ parameters for the anharmonic part of the intramolecular PES. In other words, if one wishes to determine an intramolecular PES up to quartic parameters, a total of 21 parameters is needed (or the minimum number of necessary experimentally obtained parameters is 21). Knowledge of a molecular PES with up to pentic or sextic parameters needs, as a minimum, 36 or 54 parameters, which are derived from experimental (in the present consideration, MW and SMW data) spectra. As one can see, 15 rotational, Δ–, and H–type parameters are not sufficient to obtain a molecular PES, even up to the quartic parameters. However, there are at least two additional important possibilities for the solution of this problem.

On the one hand, one can try to use the MW and SMW transitions not only of the ground vibrational state of a molecule but of the lowest isolated vibrational states as well. For the XY_2_ ($C_{2v}$) molecule, the vibrational energy of the lowest excited vibrational state (namely, (010)) is high enough. However, for molecules with a larger number of nuclei, the vibrational energies of not only one but of some of the lowest isolated vibrational states are low enough, and corresponding MW and SMW spectra can be experimentally recorded (as an illustration, we can note that, for the above-mentioned CH_2_Cl_2_ molecule, the MW and SMW spectra were recorded and analyzed not only for the ground vibrational state but for the excited states $\left(v_{4}=1\right)$, $\left(v_{4}=2\right)$, and $\left(v_{3}=1\right)$ as well; see, e.g., [29,30]). This means that the number of experimentally determined parameters can be increased by at least four times, i.e., up to 60.

The second possibility to increase the amount of initial experimental information is the use of the MW and SMW spectra of different isotopologues of a molecule (in particular, for the mentioned ethyl chloride and vinyl chloride molecules, spectra of more than ten different isotopologues were recorded in [20] and used for the determination of the structural parameters of these molecules). In this case, the addition of any vibrational state of any new isotopologue can increase the number of initial experimental parameters by fifteen. Returning to the XY_2_ ($C_{2v}$) molecule, we can see that the consideration of one additional isotopologue can increase the number of experimental parameters to up to thirty. The latter means that experimental information about only two different isotopologues of such a molecule is sufficient to derive highly accurate values for the PES parameters up to the quartic level (in general, 21 parameters from the values of 30 experimental parameters, e.g., *B*, Δ, and *H*). In turn, the MW and SMW spectra of three different isotopologues of a discussed molecule (i.e., 45 experimental parameters) allow us to determine 36 basic parameters of a molecule up to the pentic anharmonic ones. To determine 54 basic parameters, one can try to use experimental information about four different isotopologues. In general, the higher the number of used isotopologues, the higher accuracy of the derived result.

It would be interesting to consider the following. Of course, one can try to use the intramolecular PES parameters (which are derived from MW and SMW experimental data) for the estimation of the rotational/rotational–vibrational energy structure not only in the MW and SMW regions but in the IR and even visible regions. To illustrate such a possibility, we used the values of the determined $f_{n}$ parameters ($f_{2}=5.1458$ md/Å, $f_{3}=-6.0878$ md/Å, $f_{4}=9.2504$ md/Å, and $f_{5}=-10.8379$ md/Å) of the potential energy surface in the form of Equation (11) for the estimation of the line positions of the H^35^Cl molecule in its fundamental and first three overtone ro-vibrational bands. As the analysis showed, the band centers of the fundamental band and the first three lowest overtone bands were predicted with accuracy of 1–4 cm^−1^; the rotational energies (J≤10) were predicted with accuracy of 0.1 cm^−1^ (evidently, the accuracy of both the “vibrational” and “rotational” predictions can be improved by adding SMW data about the TCl molecule). In summary, one can conclude that the suggested method of intramolecular PES determination allows one to predict, with sufficiently high accuracy, the rotation–vibration energy structure in wide parts of the infrared (and probably visible) region on the basis of a very limited amount of highly accurate experimental information about the positions of the lines from the MW and SMW regions (for the HCl molecule, which was considered in the present study, only fifteen lines of H^35^Cl and D^35^Cl were used). One can expect that an analogous conclusion could be made concerning polyatomic molecules, which will be considered by the authors in the near future.

## 6. Conclusions

We have suggested a new method for the precise semiempirical determination of the basic parameters (structural parameters and parameters of the intramolecular potential energy surface, PES) of a molecule on the basis of highly accurate experimental information from its microwave and submillimeter-wave spectra. The possibilities and advantages of the suggested method in comparison with other methods of molecular PES determination were discussed, with a diatomic molecule as a relevant example. As an illustration, the HCl molecule was considered, and a comparison was performed with the results obtained on the basis of the semiempirical PES parameters that were determined from IR experimental data.

## Figures and Tables

**Table 1:** Experimental transitions of the H^*M*^Cl and D^*M*^Cl (M=35,37) species (in MHz ^(*a*)^).

J–J′	H^35^Cl ^(*b*)^	H^37^Cl ^(*b*)^	D^35^Cl ^(*c*)^	D^37^Cl ^(*c*)^
1	2	3	4	5
1–0	625,915.20 (10)	624,975.04 (10)		
2–1	1,251,450.48 (20)	1,249,571.39 (20)		
3–2	1,876,226.60 (20)	1,873,410.07 (20)		
4–3	2,499,864.42 (20)	2,496,115.33 (20)		
5–4	3,121,986.49 (20)	3,117,308.72 (20)		
6–5	3,742,216.58 (25)	3,736,615.72 (25)		
7–6	4,360,179.99 (25)	4,353,662.88 (25)	2,257,437.630 (40)	2,250,847.632 (60)
8–7	4,975,504.67 (30)	4,968,078.97 (30)		
9–8	5,587,820.10 (40)	5,579,495.42 (30)	2,897,601.923 (40)	2,889,157.278 (65)
10–9	6,196,759.71 (35)	6,187,546.55 (40)		
12–11			3,850,850.810 (50)	3,839,665.379 (80)
15–14			4,793,354.621 (40)	4,779,490.716 (70)
17–16			5,414,435.487 (93)	5,398,828.348 (89)

^(*a*)^ Values in parentheses are 1σ statistical confidence intervals. ^(*b*)^ Reproduced from Ref. [26]. ^(*c*)^ Reproduced from Ref. [27].

**Table 2:** Values of the $B^{\left(0\right)}$, $D^{\left(0\right)}$, and $H^{\left(0\right)}$ spectroscopic parameters of the ground vibrational state of the H^*M*^Cl and D^*M*^Cl (M=35,37) molecules (in MHz ^(*a*)^).

Parameter	H^35^Cl	H^37^Cl	D^35^Cl	D^37^Cl
1	2	3	4	5
$B^{\left(0\right)}$	312,989.238565 (78)	312,519.06730 (73)	161,656.210251 (34)	161,183.084353 (61)
$D^{\left(0\right)}$	−15.830367011 (26)	−15.78294216 (24)	−4.1954032453 (13)	−4.1707443247 (23)
$H^{\left(0\right)}\times 10^{4}$	4.9239727242 (75)	4.931377581 (71)	0.671134032574 (41)	0.664701939630 (73)

^(*a*)^ Values in parentheses are 1σ statistical confidence intervals.

**Table 3:** Values of the $Y_{ij}$ spectroscopic parameters of the ground vibrational states of the H^*M*^Cl and D^*M*^Cl (M=35,37) molecules (in MHz ^(*a*)^).

Parameter	tw I ^(*b*)^	tw II ^(*c*)^	IR [28] ^(*d*)^
1	2	3	4
$Y_{01}$	317,531.462 (42)	317,540.962 (44)	317,580.970
$Y_{11}$	−9084.37 (19)	−9129.89 (20)	−9208.96
$Y_{02}$	−15.9217514 (42)	−15.9238945 (42)	−15.94584
$Y_{12}$	0.182347 (17)	0.192615 (17)	0.22549
$Y_{03}\times 10^{-4}$	4.9090547 (76)		5.207
$Y_{13}\times 10^{-6}$	5.9545 (31)		19.44

^(*a*)^ Values in parentheses are 1σ statistical confidence intervals. ^(*b*)^ The abbreviation “tw” means “this work”. The corresponding parameter is obtained with the use of only parameters $B^{\left(0\right)}$ and $D^{\left(0\right)}$ from Table 2. ^(*c*)^ Obtained with the corrections to the values of the $B^{\left(0\right)}$ and $D^{\left(0\right)}$ parameters (see text for details). ^(*d*)^ Reproduced from Ref. [28].

**Table 4:** Values of the basic parameters of the HCl molecule.

Parameter	tw I ^(*b*)^	tw II ^(*c*)^	IR [28] ^(*d*)^
1	2	3	4
$B_{e}$ ^(*a*)^	10.5917095 (14)	10.5920264 (15)	10.5933609
ω ^(*a*)^	2991.53452 (99)	2991.4674 (10)	2991.0728
$a_{1}$ ^(*e*)^	−2.3659 (59)	−2.3661 (51)	−2.3664
$a_{2}$ ^(*e*)^	3.5599 (23)	3.5953 (16)	3.6759
$a_{3}$ ^(*e*)^	−4.1123 (98)	−4.2123	−4.7009

^(*a*)^ In cm^−1^. ^(*b*) (*c*) (*d*)^ See footnotes ^(*b*) (*c*) (*d*)^ to Table 3. ^(*e*)^ Dimensionless value.

## Data Availability

Data are contained within the article.

## Appendix A. Some *Y_ij_* Spectroscopic Parameters of a Diatomic Molecule

(A1) $$\begin{array}{c} Y_{01}=B_{e}, \end{array}$$

(A2) $$\begin{array}{c} Y_{11}=6B_{e}^{2}/\omega_{e}\left(1+a_{1}\right), \end{array}$$

(A3) $$\begin{array}{c} Y_{02}=-4B_{e}^{3}/\omega_{e}^{2}, \end{array}$$

(A4) $$\begin{array}{c} Y_{12}=-\left(12B_{e}^{4}/\omega_{e}^{3}\right)\left(19/2+9a_{1}+9a_{1}^{2}/2-4a_{2}\right), \end{array}$$

(A5) $$\begin{array}{c} Y_{03}=16B_{e}^{5}\left(3+a_{1}\right)/\omega_{e}^{4}, \end{array}$$

(A6) $$\begin{array}{c} Y_{13}=\left(12B_{e}^{6}/\omega_{e}^{5}\right)\left(233+279a_{1}+189a_{1}^{2}+63a_{1}^{3}-88a_{1}a_{2}-120a_{2}+80a_{3}/3\right). \end{array}$$

We would like to note that the parameters $a_{n}$ in Equations (19)–(24) are connected with the $k_{n}$ parameters from Equation (3) by the relations

(A7) $$\begin{array}{c} k_{n}={\left(\frac{2B_{e}}{\omega }\right)}^{n/2}\left(\frac{a_{0}}{4\pi^{2}}\right)a_{n-2} \end{array}$$
